# Inulin supplementation prior to mild traumatic brain injury mitigates gut dysbiosis, and brain vascular and white matter deficits in mice

**DOI:** 10.3389/frmbi.2022.986951

**Published:** 2022-11-30

**Authors:** Lucille M. Yanckello, Ya-Hsuan Chang, McKenna Sun, George Chlipala, Stefan J. Green, Zhentian Lei, Aaron C. Ericsson, Xin Xing, Tyler C. Hammond, Adam D. Bachstetter, Ai-Ling Lin

**Affiliations:** ^1^ Sanders Brown Center on Aging, University of Kentucky, Lexington, KY, United States; ^2^ Department of Pharmacology and Nutritional Sciences, University of Kentucky, Lexington, KY, United States; ^3^ Research Informatics Core, University of Illinois Chicago, Chicago, IL, United States; ^4^ Genomics and Microbiome Core Facility, Rush University, Chicago, IL, United States; ^5^ Metabolomics Center, University of Missouri, Columbia, MO, United States; ^6^ Department of Biochemistry, University of Missouri, Columbia, MO, United States; ^7^ Department of Veterinary Pathobiology, University of Missouri, Columbia, MO, United States; ^8^ Department of Computer Science, University of Kentucky, Lexington, KY, United States; ^9^ Department of Neuroscience, University of Kentucky, Lexington, KY, United States; ^10^ Spinal Cord and Brain Injury Research Center, University of Kentucky, KY, United States; ^11^ Department of Radiology, University of Missouri, Columbia, MO, United States; ^12^ Institute for Data Science &Informatics, University of Missouri, Columbia, MO, United States; ^13^ Department of Biological Sciences, University of Missouri, Columbia, MO, United States

**Keywords:** prebiotics, inulin, traumatic brain injury, microbiome, short chain fatty acids, MRI, cerebral blood flow, white matter integrity

## Abstract

**Introduction:**

Mild traumatic brain injury (mTBI) has been shown to negatively alter bacterial diversity and composition within the gut, known as dysbiosis, in rodents and humans. These changes cause secondary consequences systemically through decreased bacterial metabolites such as short chain fatty acids (SCFAs) which play a role in inflammation and metabolism. The goal of the study was to identify if giving prebiotic inulin prior to closed head injury (CHI) could mitigate gut dysbiosis, increase SCFAs, and improve recovery outcomes, including protecting cerebral blood flow (CBF) and white matter integrity (WMI) in young mice.

**Methods:**

We fed mice at 2 months of age with either inulin or control diet (with cellulose as fiber source) for two months before the CHI and continued till the end of the study. We analyzed gut microbiome composition and diversity, determined SCFAs levels, and measured CBF and WMI using MRI. We compared the results with Naïve and Sham-injury mice at 24 hours, 1.5 months, and 3-4 months post-injury.

**Results:**

We found that both CHI and Sham mice had time-dependent changes in gut composition and diversity after surgery. Inulin significantly reduced the abundance of pathobiont bacteria, such as E. coli, Desulfovibrio spp and Pseudomonas aeruginosa, in Sham and CHI mice compared to mice fed with control diet. On the other hand, inulin increased SCFAs-producing bacteria, such as Bifidobacterium spp and Lactobacillus spp, increased levels of SCFAs, including butyrate and propionate, and significantly altered beta diversity as early as 24 hours post-injury, which lasted up to 3-4 months post-injury. The mitigation of dysbiosis is associated with protection of WMI in fimbria, internal and external capsule, and CBF in the right hippocampus of CHI mice, suggesting protection of memory and cognitive functions.

**Discussion:**

The results indicate that giving inulin prior to CHI could promote recovery outcome through gut microbiome modulation. As inulin, microbiome analysis, and MRI are readily to be used in humans, the findings from the study may pave a way for a cost-effective, accessible intervention for those at risk of sustaining a head injury, such as military personnel or athletes in contact sports.

## 1 Introduction

Brain injury impacts approximately 42 million people worldwide every year (Gardner and Yaffe, 2015), and a wide range of injury severities and types are classified as mild traumatic brain injury (mTBI), including those sustained by military personnel and athletes in contact sports. Center for Disease Control and Prevention (CDC) reporting that around 5.3 million people live with a permanent disability after injury (Center for Disease Control and Prevention, 2003) and currently there are no effective therapeutics for mTBI. Therefore, development of safe and effective treatments is critical to promote recovery and rehabilitation for the millions who suffer a mTBI each year (Furlan et al., 2020).

The microbiome-gut-brain axis has emerged as a topic of interest in mTBI research (Treangen et al., 2018; Yanckello et al., 2022). Considerable evidence demonstrates that mTBI with a closed head injury (CHI) in animal models increased intestinal permeability and gut dysbiosis as early as 24 hours post-injury, which persists for as long as 3 months (Treangen et al., 2018; Yanckello et al., 2022). CHI disrupts microbiome diversity, increases pathobiont bacteria and decreases putatively beneficial bacteria (Treangen et al., 2018; Nicholson et al., 2019). CHI also has an impact on cerebral blood flow (CBF) (Bonne et al., 2003; Lyons et al., 2018) and white matter integrity (WMI) (Messe et al., 2011), which are typical chronic phenotypes of traumatic brain injury in humans.

Emerging evidence shows that manipulation of the gut microbiome through dietary intervention may be actionable in reducing long-term symptoms after mTBI (Yu et al., 2019). In a recent study, we showed that prebiotic inulin, a non-digestible dietary fiber, increased the level of beneficial bacteria, reduced pathobiont bacteria, and elevated SCFAs in the cecal contents and plasma during the chronic phase of TBI-recovery (Yanckello et al., 2022). In another study, we also showed that increased in beneficial bacteria was associated with increased CBF (Ma et al., 2018). However, it still remains unclear whether modulation of the gut microbiome *prior to injury* would have a better protection for gut microbiome and brain physiology, especially having a positive impact on CBF and WMI. Therefore, the goal of the study was to fill the knowledge gap. The outcomes from the study may provide useful and important information to better protecting brain functions for those who are at high risk for TBI, such as military personnel and athletes in contact sports.

## 2 Materials and methods

### 2.1 Experimental design

Male C57BL/6 wild-type mice were used in the study. They were randomly assigned to one of the following six groups: Naïve-Control (n =11), Sham-Control (n = 12), CHI-Control (n = 12), Naïve-Inulin (n = 12), Sham-Inulin (n = 12) and CHI-Inulin (n = 12). The number of animals per group was determined *via* power analysis to ensure comparison with a = 0.05 and b = 0.20 for detecting a true difference of each measured variable between the groups. The Control groups were fed with a diet containing cellulose that is nonfermentable (TestDiet catalog number 1817794 -203). The inulin diet was also from TestDiet (catalog number 1817795-203). **Table 1** shows the diet composition. We fed the mice 8% inulin because 8% inulin has shown positive results on the gut microbiome and related metabolites and other metrics such as brain and systemic metabolism in previous studies from our lab (Hoffman et al., 2019; Yanckello et al., 2021). Inulin has a higher percentage of carbohydrates than cellulose because inulin is fermentable fiber. This means that inulin can be fermented by the gut microbiome and turned into metabolites such as SCFAs, which are energy sources for the body (Dahl and Stewart, 2015). Mice had *ad libitum* access to the diets, and we measured the weight of the mice and the weight of the remaining food biweekly to estimate the food intake during the feeding period.

**Table 1:** Diet Composition. Comparison of diet composition of the Prebiotic (inulin) diet and Control (cellulose) diet.

Diet	Prebiotic Diet	Control Diet
Protein %	18.2	18.2
Carbohydrate %	59.1 (w/o inulin) 8.9 from inulin	60.2 (w/o inulin)
Fat %	7.1	7.1
Fiber %	8.0 (Inulin)	8.0 (Cellulose)
Energy (kcal/g)	4.08	3.78

**Figure 1:**
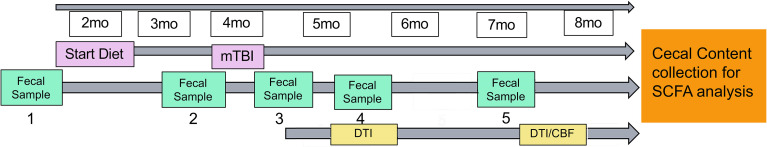
Study design. Mice started diet (control or inulin) 2 months prior to injury at 2 months of age. Fecal samples were taken periodically throughout the study at (1) 2 months of age (prior to diet administration), (2) 24 hours prior to injury, (3) 24 hours post-injury, (4) 1.5 months post-injury, and (5) 3 months post-injury. Diffusion tensor imaging was taken at 1.5 months post-injury and 3-4 months post-injury to determine white matter integrity. Arterial spin labeling was taken 3-4 months post-injury to determine cerebral blood flow.

**Figure 1** shows the study design. Mice were administered a CHI at 4 months of age. We started the diet administration started at 2 months prior to injury (i.e., 2 months of age) and continue throughout the study (till 7-8 months of age). Fecal samples were collected throughout the study at 2 months of age (prior to diet administration), 24 hours before injury, 24 hours post-injury, 1.5 months post-injury and 3 months post-injury. At 1.5 months post-injury, we assessed WMI (indexed by fractional anisotropy (FA)). At 3-4 months post-injury, we assessed WMI and CBF using MRI. We targeted to finish all the experiments when the mice reached 3 months post-injury (i.e., 7 months of age), but due to the pandemic, the MRI experiment has been delayed for several mice. Nonetheless, we managed to complete all scans before the mice reach to 8 months of age. As soon as completing the MRI scans, mice were euthanized by carbon dioxide inhalation and decapitated. Cecal contents was collected thereafter for SCFAs analyses. Mice were singly housed throughout the study to avoid feces exchange (as mice are coprophagic) in an AAALAC accredited/PHS facility under specific pathogen-free conditions. All experimental procedures were performed according to NIH guidelines and approved by the Institutional Animal Care and Use Committee (IACUC) at the University of Kentucky (UK).

### 2.2 Closed head injury

Briefly, as previously described (Bachstetter et al., 2015; Webster et al., 2015), mice were anesthetized with isoflurane (4-5% induction followed by 2-3% maintenance) prior to insertion of ear bars to stabilize the head in the digital mouse stereotaxic frame (Stoelting Co., Chicago, IL). A midline sagittal scalp incision was made, and a 1ml latex bulb (Fisher Scientific) was placed under the head and filled with water to displace the force during impact from the ears. An electromagnetic impactor was used to deliver a single controlled mid-line cortical impact at 5.0 ± 0.2 m/s, 100ms dwell time, and 1.0 ± 0.2 mm impact depth. Planned exclusion criteria included depressed skull fracture or bleeding; however, no mice were excluded from the study. Sham-injured mice underwent identical surgical procedures, but no impact was delivered. Naïve mice did not undergo any surgical procedures or anesthetic administration. Righting-reflex was recorded as an acute neurological assessment. Within one hour after injury, animals were fully conscious and able to ambulate.

### 2.3 Gut microbiome sequencing and analysis

Fecal samples were collected in a two-hour window between 7-9 am. Samples were frozen at -80°C until DNA extraction. Genomic DNA was extracted from fecal samples and ZymoBIOMICS™ DNA Mini Kit per manufacturers guidelines. According to the manufacturer’s instructions, genomic DNA (20 ng input) was used as template for shotgun metagenome library preparation using the Illumina DNA Prep (formerly Nextera FLEX) protocol. DNA was subject to tagmentation and then amplified using 10 cycles of PCR to incorporate unique dual indices. The amplified products were cleaned, and size selected by two incubations with SPB at 0.53X and 0.12X and eluted in water. Libraries were pooled and initially sequenced on an Illumina MiniSeq mid-output flowcell as part of library QC. Based on the output from the MiniSeq run, samples were re-pooled to produce even sequence yields. The final pool was sequenced on an Illumina NovaSeq6000 instrument using an S4 flow cell and paired-end 2×150 sequencing reads. Library preparation and QC sequencing were performed at the Genomics and Microbiome Core Facility (GMCF) at Rush University. NovaSeq sequencing was performed at the W.M. Keck Center for Comparative and Functional Genomics at the University of Illinois at Urbana-Champaign (UIUC). The gene amplicon sequence data generated as part of this study have been submitted to the NCBI BioProject database (PRJNA903182).

For taxonomic annotation, raw reads were mapped to the NCBI nucleotide database using Centrifuge (Kim et al., 2016). Taxonomic annotations for each read were obtained using least common ancestor algorithm and then summarized across all reads to create counts per taxon. Raw counts were normalized to percentages for relative abundance.

Differential analyses of bacterial taxa as compared with experimental covariates (i.e., injury, diet) were performed using the software package edgeR on raw sequence count (Robinson et al., 2010). Prior to analysis, the data were filtered to only include those taxa that were annotated as Bacteria but were not annotated as chloroplast or mitochondria in origin as well as removing taxa removed any taxon that had less than 1000 total counts across all samples and were present in less than 20% of the samples. Data were normalized as counts per million. Normalized data were then fit using a negative binomial generalized linear model using experimental covariates, and statistical tests were performed using a likelihood ratio test. Adjusted p values (q values) were calculated using the Benjamini-Hochberg false discovery rate (FDR) correction (Benjamini and Hochberg, 1995). Significant taxa were determined based on an FDR threshold of 5% (0.05).

### 2.4 Diversity and dissimilarity analyses

#### 2.4.1 Alpha diversity analyses

Shannon indices were calculated with default parameters in R using the vegan library (Oksanen et al., 2018). Prior to analysis, the data were filtered to only include those taxa that were annotated as Bacteria but were not annotated as chloroplast or mitochondria and then rarefied to a depth of 800,000 counts per sample. The resulting Shannon indices were then modelled with the sample covariates using a generalized linear model (GLM) assuming a Gaussian distribution. Significance of the model (ANOVA) was tested using the F test. *Post-hoc*, pairwise tests were performed using Mann-Whitney test. Plots were generated in R using the ggplot2 library (Wickham, 2009).

#### 2.4.2 Beta diversity/dissimilarity analyses

Bray-Curtis indices were calculated with default parameters in R using the vegan library (Oksanen et al., 2018). Prior to analysis the normalized data were square root transformed. The resulting dissimilarity indices were modelled and tested for significance with the sample covariates using the ADONIS test. Additional comparisons of the individual covariates were also performed using ANSOIM. Plots were generated in R using the ggplot2 library (Wickham, 2009).

### 2.5 MRI data acquisition

We used the 7T Bruker BioSpin MRI GmbH Scanner (Siemens, Germany) at the Magnetic Resonance Imaging & Spectroscopy Center of UK. Mice were anesthetized with 4.0% isoflurane for induction and then maintained in a 1.2% isoflurane and air mixture using a facemask. Heart rate (90–130 bpm.), respiration rate (70-90 breaths per minute), and rectal temperature (37 ± 0.5°C) were monitored. A water bath with circulating water at 45–50°C was used to maintain the body temperature. Qualitative CBF was measured using arterial spin labeling method (Fair Rare perfusion weighted sequence) in an interleaved fashion (TI loop outside) with the following parameters: FOV = 20 x 20 mm^2^, matrix = 128 x 112, slice thickness = 1 mm, 1 slice, TR = 10000 ms, TE = 44 ms, echo spacing = 5.5 ms, rare factor = 16, resolution = 0.156 x 0.179, anti-aliasing = 1 x 1, excitation angle = 90 degrees, refocusing angle = 180 degrees, 8 repetitions, TI values = 20 ms with constant recovery; calculated inversion pulse with 4 mm inversion slab thickness and 1.5 mm slice package margin. CBF was visualized and quantitated using Mango image viewer (UT San Antonio, TX). Diffusion tensor imaging (DTI) is used to characterize microstructural changes in the brain (Alexander et al., 2007). The images are acquired using the Bruker DTI : EPI, SpinEcho sequence with the following parameters: TE = 0.512 ms, TR = 2000 ms, diffusion time = 8.4 ms, encoding direction 2.5 ms. A DTI diffusion scheme was used with a total of 40 sampling direction acquired, b-value = 672.39 s/mm^2^, in plane resolution = 0.179688 mm, slice thickness 0.8 mm. Tensor metrics were calculated using DWI with b-value lower than 1750 s/mm^2^. Fractional Anisotropy (FA) values were analyzed using DSI studio (Yeh et al., 2019).

### 2.6 Short-chain fatty acids of cecal contents

Samples were prepared for LCMS by adding 200 µL of isopropyl alcohol containing an internal standard 2-isobutoxyacetic acid (15 µg/mL) to 20 mg of cecal contents in a 1.5 mL microcentrifuge tube to reach a mass to volume ratio of 0.1 mg/µL. Samples were shaken for 30 min and then centrifuged for 30 min at 13,000×g. 50 µl of the supernatant was transferred to a glass sample vial insert and derivatized according to previously published method with some modifications (Dei Cas et al., 2020). Briefly,10 μL of 100 mM of 3-NPH, 10 μL of 100 mM of EDC, 10 μL of pyridine (15%) in methanol were consequently added to the insert and capped tightly. Derivatization was performed in an incubator at 37 °C for 45 minutes. Standards (acetic acid, propionic acid, butyric acid, isobutyric acid, valeric acid, isovaleric acid and 2-methylbutanoic acid at 4 µg/mL) were also derivatized in the same manner as a positive control. The solution (2 µL) was then directly injected into a Waters Acquity UPLC system coupled to a Bruker Impact II quadrupole time-of-flight mass spectrometer (qTOF-MS). Chromatographic separation was achieved on a Waters C18 2.7 µm × 150 mm using mobile phase (A) water +0.1% formic acid and (B) acetonitrile with the following gradient: B increased from 10% to 50% over 10 min, then to 95% in 2 min, held at 95% for 2 min and returned to 10% for equilibrium. The flow rate was 0.35 mL/min and the column temperature was 35 °C. Mass spectral data were acquired in negative ion mode with a scan range from m/z 50 to m/z 500. Capillary voltage was 4.5kV, nebulizer gas pressure 44 psi, dry gas flow 12 l/min, dry gas temperature 250 °C. Short chain fatty acids were identified by comparing their retention time and m/z with those of authentic standards.

### 2.7 Statistical analysis

All statistical analyses were completed using R 3.6.2 and GraphPad Prism 9.0 (GraphPad, San Diego, CA, USA). Two-way ANOVAs were performed for determination of differences between groups. Based on the a-priori hypothesis constructed from our pilot study data, we expected a specific pattern of responses, leading us to plan a set of pairwise comparisons (Naïve control vs. Inulin, Sham control vs. Inulin, CHI control vs inulin) to assess dietary effects. These planned comparisons were reported in addition to ANOVA results (Prism, 2009; Wales, 2020). Planned comparisons were carried out using Welch’s t-test. Levels of statistical significance were reached when p < 0.05. For the microbiome, given the multiple comparisons inherent in analysis, between-group relative differences are assessed using both p-value and false discovery rate analysis (q-value).

## 3 Results

### 3.1 Inulin altered alpha and beta diversity in Sham and CHI mice

**Figure 2:**
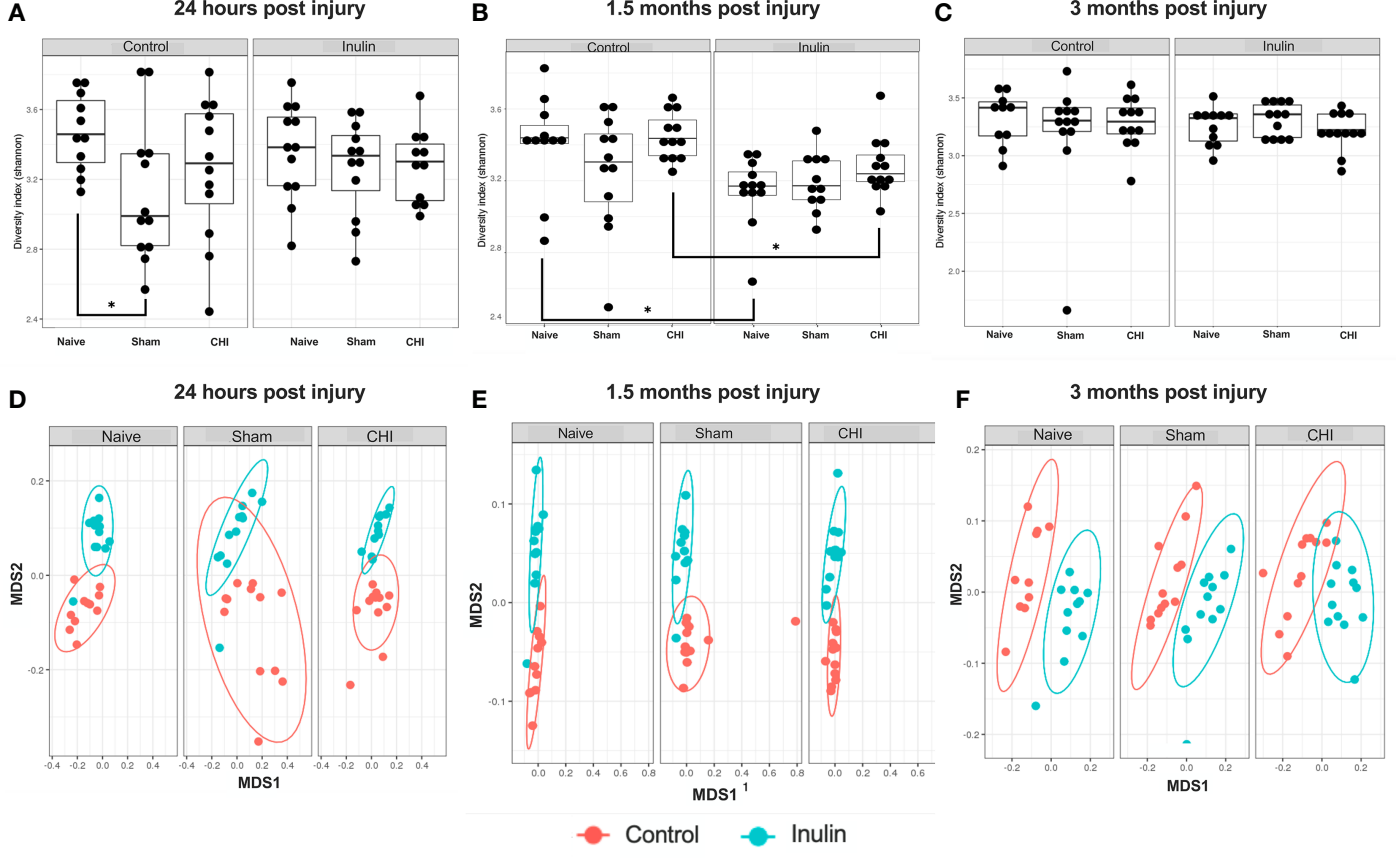
Alpha diversity and beta diversity analysis 24 hours, 1.5 months, and 3 months post-injury. Shannon index is used for all alpha diversity analyses, and Bray-Curtis index is used for all beta diversity analyses. Alpha diversity at **(A)** 24 hours, **(B)** 1.5 months, and **(C)** 3 months post-injury. Beta diversity at **(D)** 24 hours, **(E)** 1.5 months, and **(F)** 3 months post-injury. *p < 0.05.

Sham-control mice had a lower alpha diversity at 24 hours post-injury compared to the Naïve and CHI mice fed with control diet. The difference was not found in the shame-inulin mice (**Figure 2A** **)**. Alpha diversity was different between control and inulin groups for Naïve (p = 0.013) and CHI mice (p = 0.033) by 1.5 months post-injury (**Figure 2B**) with an overall diet effects among groups (F(1,66) = 4.437, p = 0.0391). There were no differences in alpha diversity regardless of diet by 3-4 months post-injury (**Figure 2C**).

Beta diversity (Bray-Curtis index) showed a time-dependent change, with increased diversity over time. It showed an overall effect due to diet at 24 hours post-injury (**Figure 2D**) (Sum of Squares = 0.213789, R2 = 0.194, F = 19.9148, p = 0.001) with pairwise comparisons showing significant differences between Naïve control and Naïve inulin mice (p = 0.001), Sham control and Sham inulin mice (p = 0.001) and CHI control and CHI inulin mice (p = 0.001). At 1.5 months post injury (**Figure 2E**), there was an overall effect due to diet (Sum of Squares = 0.34854, R2 = 0.331, F = 34.352, p = 0.001) with all three groups exhibiting significant differences between control and inulin groups (Naïve p = 0.001, Sham p = 0.001, CHI p = 0.001). By 3-4 months post injury (**Figure 2F**), beta diversity continues to show an overall effect due to diet (Sum of Squares = 0.30966, R2 = 0.386, F = 41.1556, p = 0.001) with pairwise analyses again indicating significant differences between control fed and inulin fed mice in all three groups (Naïve p = 0.001, Sham p = 0.001, CHI p = 0.001).

### 3.2 Inulin decreased pathobiont bacteria and increased beneficial bacteria throughout the all study time-points

**Figure 3:**
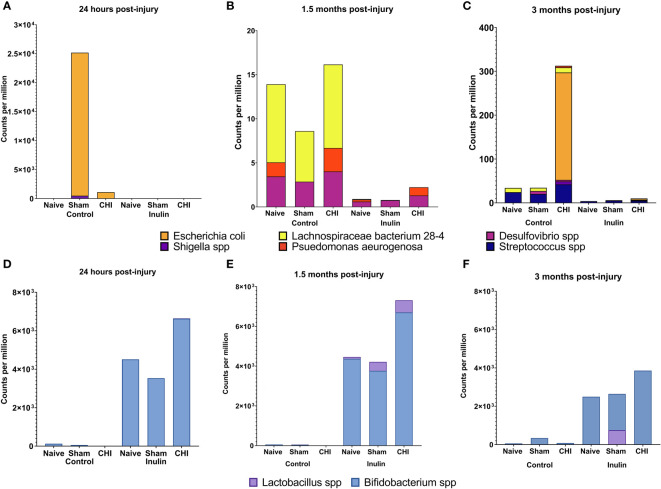
Analysis of pathobiont bacteria and beneficial bacteria at 24 hours, 1.5 months and 3 months post-injury. **(A)** At 24 hours post-injury, control-fed mice show higher levels of (*E*) *coli* and *Shigella spp* than inulin-fed mice in Sham and CHI groups. **(B)** At 1.5 months post-injury, control-fed mice show higher levels than inulin-fed mice of *L. bacterium 28-4 and Desulfovibrio spp* in Naïve, Sham, and CHI mice and higher levels of *P. aeruginosa* in Naïve and CHI mice. **(C)** At 3 months post-injury, there are higher levels in control-fed mice than inulin-fed mice of *L. bacteria 28-4* and *Streptococcus spp* in Naïve, Sham, and CHI mice, higher levels of *Desulfovibrio spp* in Sham and CHI mice, and higher levels of (*E*) *coli, Shigella spp*, and *P. aeruginosa* in CHI mice. **(D)** At 24 hours post-injury, inulin-fed mice show higher levels than control-fed mice of *Bifidobacterium spp* in Naïve, Sham, and CHI groups and higher levels of *Lactobacillus spp* in CHI mice. **(E)** At 1.5 months post-injury, inulin-fed mice show higher levels than control-fed mice of *Bifidobacterium spp* in Naïve, Sham, and CHI groups and higher levels of *Lactobacillus* spp. in Sham and CHI groups. **(F)** At 3 months post-injury, inulin-fed mice show higher levels than control-fed mice of *Bifidobacterium* spp. in Naïve, Sham, and CHI groups and higher levels of *Lactobacillus spp* in Sham and CHI groups.

We analyzed the microbiome composition across the three time points. We found that there was a time-dependent changes on microbiome composition among the Naïve, Sham and CHI mice. At 24 hours post-injury (**Figure 3A**), *Escherichia coli* (*E. coli*) and *Shigella spp* were significantly increased in Sham-Control mice (*E. coli* Q = 4.15e-08*, S. dysenteriae* Q = 2.14e-04*, S. flexneri* Q = 2.66e-05*, S. boydii* Q = 2.91e-06) and CHI-Control mice (*E. coli*, Q = 1.86e-08*, S. dysenteriae* Q = 1.39e-04*, S. flexneri* Q = 0.04*, S. boydii* Q = 2.02e-05). Interestingly, the Sham-Control mice had significantly higher magnitude of the increase in *E. coli* compared with the CHI-Control mice.

At 1.5 months post-injury (**Figure 3B**), we found a different set of pathobiont bacteria in the Control groups. In particular, *Desulfovibrio spp* and *Lachnospiraceae bacterium 28-4* were significantly higher among all the three Control groups (*L bacterium 28-4* Q = 9.01e-08*, D. other Q =* 0.02). The Naive and CHI mice had additional higher levels of *Pseudomonas aeruginosa* (*L bacterium 28-4* Q = 6.33E-19*, D. piger* Q = 5.01e-03*, D. other* Q = 0.01, *P. aeruginosa* Q = 0.02). At 3 months post-injury (**Figure 3C**), CHI-Control mice had a dramatically higher levels of *E. coli spp, Streptococcus spp, Shigella spp*, and *P. aeruginosa*, which were not seen in the Sham or Naïve mice (*L. bacterium 28-4* Q = 3.26E-17*, S. pyogenes* Q = 5.45e-05, *D. piger* Q = 4.42e-04*, D. other* Q = 0.03*, E. coli Q =* 1.79e-03*, S. dysenteriae* Q = 0.04*, S. other Q =* 2.14e-12*, S. boydii* Q = 0.02*, P. aeruginosa* Q = 1.67e-05).

Interestingly, inulin was able to reduce the levels of the all pathobiont bacteria throughout the three time-points (**Figure 3, A-C**). In contrast, inulin increased beneficial bacteria in all three groups of mice as early as 24 hours post-injury and persist until 3 months post-injury.

At 24 hours post-injury (**Figure 3D**), inulin fed mice showed higher levels than control fed mice of *Bifidobacterium spp* in Naïve (*B. other Q =* 4.19e-04), Sham (*B. other* Q = 2.89e-06*, B. pseudolongum* Q = 3.48e-04) and CHI (*B. other* Q = 1.63E-23, *B. pseudolongum* Q = 1.34E-34) and higher levels of *Lactobacillus spp* in CHI mice (*L. gasseri* Q = 5.14e-03).

At 1.5 months post-injury (**Figure 3E**), inulin fed mice showed higher levels than control fed mice (similar to that of 24 hours post injury) of *Bifidobacterium spp* in Naïve (*B. other* Q = 1.35e-05*, B animalis* Q = 1.75e-04 *and B. pseudolongum* Q = 1.37e-03), Sham (*B. other* Q = 3.98e-08*, B animalis* Q = 2.53e-07 *and B. pseudolongum* Q = 8.97e-06*, B. bifidum* Q = 1.93e-04) and CHI (*B. other* Q = 2.93E-43*, B animalis* Q = 2.43E-25 *and B. pseudolongum Q =* 1.40E-26) and higher levels of *Lactobacillus* spp. in Sham (*L. gasseri* Q = 0.01, *L. johnsonii* Q = 0.03) and CHI (*L. gasseri* Q = 7.53e-05*, L. johnsonii* Q = 1.21e-06).

At 3 months post-injury (**Figure 3F**), inulin fed mice continued to show higher levels than control fed mice of *Bifidobacterium* spp. in Naïve (*B. other* Q = 4.52e-05*, B. animals*, Q = 7.18e-05*, B. pseudolongum* Q = 4.74e-03), Sham (*B. other* Q = 1.46e-05, *B animalis* Q = 2.23e-04, *B. pseudolongum* Q = 6.07e-04, *B. bifidum* Q = 2.46e-03) and CHI (*B. other* Q = 1.46e-06*, B animalis Q =* 2.24e-06*, B. pseudolongum* Q = 3.38e-05*, B. bifidum Q =* 6.75e-03) and higher levels of *Lactobacillus spp* in Sham (*L. johnsonii* Q = 0.03) and CHI (*L. gasseri* Q = 3.75e-03).

Collectively, the data shows that there was a time-dependent changes in microbiome composition between the Naïve, Sham and CHI mice, but inulin was able to reduce the abundance of pathobiont bacteria and increase the abundance of beneficial bacteria.

### 3.3 Inulin increased cecal short-chain fatty acids levels

**Figure 4:**
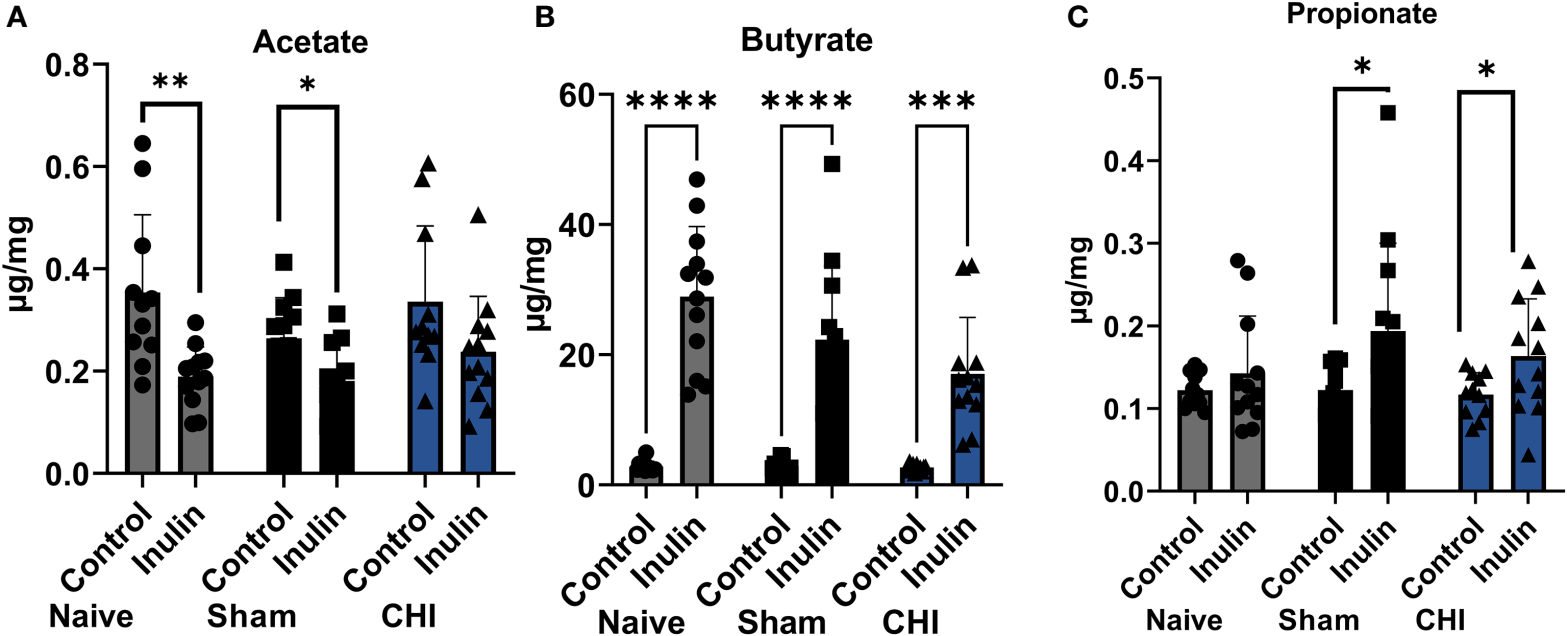
SCFAs analysis 3-4 months post-injury. **(A)** Acetate significantly increased in the control-fed mice compared to the inulin-fed mice in the Naïve and Sham groups. **(B)** Butyrate significantly increased in inulin-fed mice compared to control-fed mice in Naïve, Sham, and CHI groups. **(C)** Propionate increased in inulin-fed mice compared to control-fed mice in Sham and CHI groups. Data are mean ± SD. * p < 0.05, ** p < 0.01, *** p < 0.001, **** p < 0.0001.

All three SCFAs showed a significant effect due to diet three months post-injury. There were decreased levels of acetate (**Figure 4A** **)** in inulin-fed mice compared to control-fed mice in the Naïve (p =0.0052) and Sham mice (p = 0.0118). In contrast, we found higher levels of butyrate (**Figure 4B**) in inulin-fed Naïve (p < 0.0001), Sham (p < 0.0001) and CHI (p = 0.0001) mice compared to controls, as well as higher levels of propionate (**Figure 4C**) in inulin fed Sham (p = 0.0432) and CHI (p = 0.0482) mice compared to controls. This is consistent with our previous findings (Yanckello et al., 2022).

### 3.4 Inulin increased white matter integrity 1.5 months and 3-4 months post-injury

**Table 2** shows the WMI indexed by FA at 1.5 months post-injury. The left corpus callosum showed a significant overall effect due to diet (F(1,29) = 4.416, p = 0.0444). Although pairwise comparisons did not show statistical differences, there was a trend of inulin-fed mice exhibiting higher levels of FA compared to control-fed mice. The left external capsule also showed a significant overall effect due to diet (F(1,29) = 4.210, p = 0.0493), with pairwise comparisons showing increased FA in naïve inulin fed mice compared to control fed mice (p = 0.0362). CHI-inulin mice had significantly higher FA in right fimbria (p = 0.0012) and right external capsule (p = 0.0192) compared to CHI-control mice.

**Table 2:** Fractional anisotropy (FA) values at 1.5 months post-injury.

Groups Regions	Naïve			Sham			CHI			Diet effect F,P value
	Control	Inulin	P value	Control	Inulin	P value	Control	Inulin	P value	
Right Internal Capsule	0.5198 ± 0.0397	0.520 ± 0.0316	0.9977	0.5259 ± 0.0305	0.5407 ± 0.0241	0.4083	0.4974 ± 0.0604	0.5334 ± 0.0310	0.2010	1.604, 0.2154
Left Internal Capsule	0.4832 ± 0.0489	0.4897 ± 0.0180	0.7883	0.4877 ± 0.0444	0.5110 ± 0.0190	0.3223	0.4723 ± 0.0369	0.5104 ± 0.0491	0.1517	3.129, 0.0874
Right Fimbria	0.4431 ± 0.0580	0.4662 ± 0.0436	0.4865	0.4714 ± 0.0530	0.4596 ± 0.0318	0.6762	0.4538 ± 0.0197	0.4944 ± 0.0133	0.0012**	1.764, 0.1945
Left Fimbria	0.4311 ± 0.0404	0.4454 ± 0.0276	0.5257	0.4476 ± 0.0263	0.4347 ± 0.0388	0.5285	0.4548 ± 0.0526	0.4244 ± 0.0369	0.2493	0.5358, 0.4700
Right Corpus Callosum	0.3148 ± 0.0748	0.3120 ± 0.0355	0.9417	0.2812 ± 0.0538	0.3088 ± 0.0675	0.4696	0.3052 ± 0.0396	0.2892 ± 0.0326	0.4404	0.02767, 0.8690
Left Corpus Callosum	0.2966 ± 0.0451	0.3183 ± 0.0478	0.4595	0.2819 ± 0.0424	0.3162 ± 0.0127	0.1473	0.2752 ± 0.0263	0.2935 ± 0.0247	0.2221	4.416, 0.0444*
Right External Capsule	0.2645 ± 0.0692	0.2668 ± 0.0173	0.9467	0.2595 ± 0.0130	0.2685 ± 0.0153	0.3216	0.2488 ± 0.0198	0.2727 ± 0.0087	0.0192*	1.354, 0.2541
Left External Capsule	0.2523 ± 0.0211	0.2812 ± 0.0140	0.0362*	0.2589 ± 0.0164	0.2655 ± 0.0337	0.6836	0.2482 ± 0.0403	0.2670 ± 0.0056	0.2654	4.210, 0.0493*

Data were mean ± SD. * p < 0.05, ** p < 0.01.

**Figure 5:**
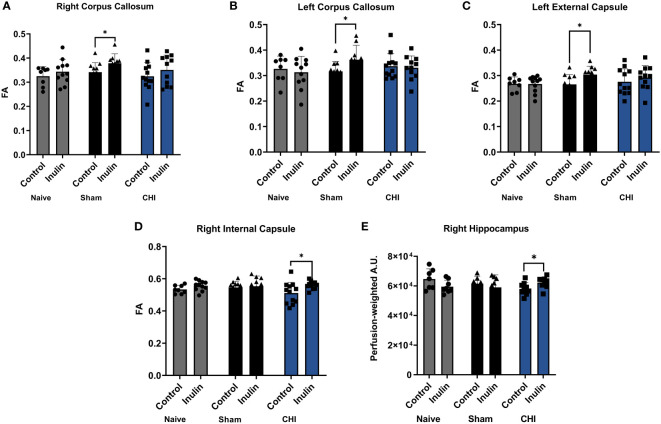
White matter integrity analysis and cerebral blood flow 3-4 months post-injury. Sham-inulin mice had higher FA than the sham-control in **(A)** right and **(B)** left corpus callosum, and **(C)** left external capsule. **(D)** CHI-inulin mice had higher FA than the CHI-control mice in the right internal capsule. **(E)** CHI-inulin mice had higher CBF in the right hippocampus than the CHI-control mice. Data are mean ± SD. *p < 0.05.

**Table 3** shows the WMI indexed by FA at 3-4 months post-injury, and **Figure 5** illustrates the regions with significant difference among groups. In the Sham mice, higher FA values were found in the right corpus callosum (p = 0.0461) (**Figure 5A**), left corpus callosum (p = 0.0481) (**Figure 5B**), and left external capsule (p = 0.0228) (**Figure 5C**) in the inulin-fed mice compared with controls. In contrast, CHI-inulin mice had higher FA values in the right internal capsule compared with CHI-control mice (p = 0.0317) (**Figure 5D**).

**Table 3:** Fractional anisotropy (FA) values 3-4 months post-injury.

Groups Regions	Naïve			Sham			CHI			Diet effect F, P value
	Control	Inulin	P value	Control	Inulin	P value	Control	Inulin	P value	
Right Internal Capsule	0.5337± 0.0254	0.5566± 0.0309	0.0953	0.5460± 0.0378	0.5531± 0.0638	0.7626	0.5112± 0.0666	0.5603± 0.0249	0.0317*	5.125, 0.0274*
Left Internal Capsule	0.5490± 0.0503	0.5355± 0.0482	0.5632	0.5206± 0.0465	0.5073± 0.0548	0.5575	0.5248± 0.0679	0.5299± 0.0312	0.8164	0.3102, 0.5797
Right Fimbria	0.4645± 0.0346	0.4528± 0.0498	0.5517	0.4630± 0.0478	0.4868± 0.0609	0.3363	0.4663± 0.0505	0.4907± 0.0287	0.1685	1.032, 0.3139
Left Fimbria	0.4799± 0.0235	0.4690± 0.0541	0.5637	0.4581± 0.0143	0.4513± 0.0551	0.7124	0.4724± 0.0566	0.4683± 0.0423	0.8437	0.4018, 0.5287
Right Corpus Callosum	0.3250± 0.0394	0.3437± 0.0509	0.3807	0.3416± 0.0396	0.3784± 0.0393	0.0461*	0.3245± 0.0573	0.3508± 0.0612	0.3006	4.690, 0.0345*
Left Corpus Callosum	0.3262± 0.0502	0.3131± 0.0626	0.6197	0.3164± 0.0389	0.3623± 0.0567	0.0481*	0.3370± 0.0495	0.3306± 0.0464	0.7510	0.4571, 0.5017
Right External Capsule	0.2681± 0.0275	0.2649± 0.0416	0.8431	0.2638± 0.0479	0.3015± 0.0516	0.0898	0.2866± 0.0433	0.2713± 0.0442	0.4118	0.3292, 0.5684
Left External Capsule	0.2679± 0.0267	0.2670± 0.0327	0.9476	0.2654± 0.0385	0.3040± 0.0327	0.0228*	0.2756± 0.0507	0.2919± 0.0470	0.4346	3.144, 0.0815

Data were mean ± SD. * p < 0.05.

### 3.5 Inulin increased cerebral blood flow (CBF) 3-4 months post-injury in CHI mice

CBF is shown as perfusion-weighted values with an arbitrary unit (A.U.). Pairwise comparisons in the right hippocampus showed a significant increase in CBF in the CHI-inulin compared to the CHI-control in the right hippocampus (p = 0.0390) (**Figure 5E**). This is consistent with our previous study that showed inulin increased CBF even when administered during the chronic recovery period of CHI (Yanckello et al., 2022). Other regional CBF values can be found in **Table 4**.

**Table 4:** Cerebral blood flow perfusion-weighted values at 3-4 months post-injury.

Groups Regions	Naïve			Sham			CHI			Diet effect F, P value
	Control	Inulin	P value	Control	Inulin	P value	Control	Inulin	P value	
Whole Brain	54603± 5995	50517± 4913	0.1786	51527± 4260	49773± 7253	0.5562	48862± 3645	51789± 3719	0.1112	0.4335, 0.5138
Left Adjacent	76263± 7979	70371± 4252	0.1147	72248± 5290	70190± 11787	0.6492	68323± 4676	73109± 5568	0.0663	0.2618, 0.6115
Right Adjacent	70152± 7432	66046± 4421	0.2318	69012± 4527	66294± 8231	0.4159	64841± 4851	68883± 4183	0.0770	0.3062, 0.5829
Left Proximal	77883± 8626	69592± 5550	0.0543	72540± 6315	70127± 10330	0.5741	67824± 5378	72449± 7063	0.1389	0.8949, 0.3494
Right Proximal	76322± 9566	69170± 5626	0.1156	72463± 5976	69225± 10280	0.4441	67750± 5097	71404± 6583	0.2077	1.099, 0.3003
Left Hippocampus	66951± 6932	62629± 6302	0.2323	63845± 4392	60913± 9538	0.4299	60370± 3850	63731± 4193	0.0957	0.5246, 0.4728
Right Hippocampus	64653± 6875	59464± 4364	0.1170	61445± 5080	59028± 8412	0.4892	58010± 4169	62218± 3752	0.0390*	0.4801, 0.4921
Left Thalamus	45757± 4151	42654± 2569	0.1185	44167± 3841	41215± 6399	0.2728	41491± 3122	44145± 3149	0.0915	0.9250, 0.3415
Right Thalamus	45474± 4640	41705± 2484	0.0872	43430± 3273	41378± 5404	0.3645	40518± 2615	43166± 2711	0.0510	0.9959, 0.3239

Data were mean ± SD. * p < 0.05.

## 4 Discussion

In this study, we demonstrate that there was a post-injury time-dependent gut microbiome composition changes in the Sham and CHI mice. It is consistent with our previous finding that Sham mice had a significantly transient increase in *E. coli* at the first 24 hours after injury and then normalized (Yanckello et al., 2022). The CHI mice experienced a different pattern of change, with higher levels of *Pseudomonas aeruginosa*, (at 1.5 months after injury), which is known to cause various life-threatening infections and alter intestinal barrier function (Laughlin et al., 2000; Moradali et al., 2017). CHI mice also had higher *Desulfovibrio spp* and *Lachnospiraceae* spp. *Desulfovibrio spp* is an intestinal pathogen that can infiltrate the central nervous system, and plays a key role in Parkinson’s Disease (Murros et al., 2021)*. Lachnospiraceae* spp are part of the *Lachnospiraceae* family, known to be associated with colitis and metabolic disorders (Nakanishi et al., 2015; Vacca et al., 2020). CHI mice showed a dramatically increased in *E. coli* at 3 months post-injury.

It is interesting to note that Sham mice also experienced gut microbiome changes in diversity and composition despite without having CHI. We had a similar observation in our previous study (Yanckello et al., 2022) and were therefore suggested to include a Naïve cohort in the current study as a negative control. It will also be important for other CHI-related studies to include a Naïve cohort in the future, especially those related to microbiome research.

We further demonstrated that inulin was able to shift the balance between the pathobiont and beneficial (SCFA-producing) microbiota at as early as 24 hours post-injury. In particular, we found increased level of *Bifidobacterium spp* and *Lactobacillus* spp. *Bifidobacterium spp* are acetate producers, while *Lactobacillus spp* are propionate and butyrate producers (Markowiak-Kopec and Slizewska, 2020). This is consistent with our SCFAs data which showed that butyrate and propionate were increased with inulin. Further, bacteria such as *Bifidobacterium pseudolongum* and *Bifidobacterium animalis* have anti-inflammatory and anti-pathogenic qualities (Servin, 2004; Riedel et al., 2006), and *Lactobacillus johnsonii* and *Lactobacillus gasseri* are known to be immunomodulators and inhibit pathogens (Servin, 2004; Wells, 2011).

Interestingly, we found that acetate was lower in the inulin-fed groups compared with controls. This could be because there are higher acetate-producing bacteria in the control-fed mice, such as commensal *Streptococcus* species. It could also be due to interconversion of SCFAs from acetate to butyrate, which occurs at a high level in the cecum (den Besten et al., 2013). Further, acetate utilization has been shown be play a key role in producing oligodendrocytes (Rosko et al., 2019; Moffett et al., 2020), which is important for protecting WMI. This is consistent with our FA findings.

Inulin increased FA of the CHI mice in the right fimbria and right external capsule at 1.5 months post-injury, and in the right internal capsule at 3-4 months post-injury. The fimbria is the major white matter output tract of the hippocampus, where damage can cause difficulty in long term recall (Dahmani et al., 2019). The external capsule is the route for cholinergic fibers from the forebrain to the cortex (Fields, 2008) while the internal capsule is an avenue for communication between the cerebral cortex and brain stem (Nolte, 2009). Decreased FA in the external capsule has repeatedly been shown after mTBI (Arfanakis et al., 2002; Inglese et al., 2005). Consequently, our findings suggest that inulin may be protective to memory and brain connectivity between brain regions.

WMI was not impacted in the Sham mice, but we observed that inulin enhanced WMI of Sham mice at 3-4 months post injury. Sham-inulin mice had higher FA in both sides of corpus callosum, and the left external capsule. The corpus callosum integrates and transfers information from both cerebral hemispheres to process sensory, motor, and cognitive signals (Goldstein et al., 2022). Traumatic brain injury has been found to cause lesions and demyelination in the corpus callosum (Goldstein et al., 2022). Our findings indicate that inulin might also benefit brain connectivity in the Sham mice.

Similar to the FA results, we found that inulin also protected CBF in the hippocampus of the CHI mice, suggesting a protection of cognitive function. This may also be associated with the changes in SCFAs, as butyrate is known to increase neurogenesis and reduce inflammation, which could be beneficial in terms of CBF and brain vascular integrity (Matt et al., 2018).

Our findings are in line with another study showing that young male mice fed with various supplements of fruit or vegetable blends prior to moderate TBI, had reduced neuroinflammation and improved functional recovery compared to controls (Yu et al., 2019). These findings indicate that pretreatment with prebiotic fiber could be effective to accelerate recovery from a mild or moderate TBI. Our findings may also have important implications for mitigating TBI-associated dementia in the future. It is well documented that TBI increases the risk for developing dementia, such as Alzheimer’s disease (AD) later in life Mortimer et al., 1991; Gavett et al., 2010; Lee et al., 2013; (Barnes et al., 2016). Protecting brain vascular and metabolic functions (Hammond et al., 2020; Hammond et al., 2021; Hammond and Lin, 2022) is critical to slowing down brain aging (Hoffman et al., 2017) and mitigating the risk for AD (Lee et al., 2018; Lin et al., 2020), as these functions serve an essential role in sustaining essential brain activities (Lin et al., 2008; Lin et al., 2010). It is also consistent with our previous findings that inulin is protective of systemic metabolism and reduces the risk for AD in a mouse model with human *APOE4* alleles, the strongest genetic risk factor for AD (Hoffman et al., 2019; Yanckello et al., 2021).

A limitation of this study is that only male mice were used. Female mice will be included in future studies to understand sex effects on injury and diet. It will also be critical to determine the underlying mechanism of CBF and WMI protection with inulin, and whether SCFAs play a key role. In this study, we reported SCFAs from the cecal content; plasma and brain SCFAs levels will also needed to be included in the future studies. Further, although we believe the transient dysbiosis in Sham mice comes primarily from anesthetic inhalation, a study that further elucidates the changes seen in Sham mice needs to be conducted. Another limitation is that the mice were singly housed in the study, which may induce stress. A recent study showed that there were no differences in exploratory activity, anxiety, working memory and fear memory processing in male C57BL/6JRj mice that were singly housed, pair housed or pair housed with a cage divider (Buckinx et al., 2021). However, the issue will be further addressed in our future studies, such as collecting fecal samples before placing the mouse into a cage individually, and perform additional anxiety tests, such as elevated plus maze or open field, to rule of the potential confounding factor induced by stress.

In summary, we found that inulin supplementation prior to injury may beneficially affect the gut microbiome by reducing pathobiont bacteria, increasing putatively beneficial bacteria, increasing SCFAs levels in cecal contents, and protecting WMI and CBF in CHI mice. As inulin, microbiome analysis, and MRI are readily to be used in humans, giving prebiotic fiber could be a cost-effective, accessible intervention for those at risk of sustaining a head injury, such as military personnel or athletes in contact sports.

## 5 Acknowledgements

The content is solely the responsibility of the authors and does not necessarily represent the official views of the National Institute on Aging or the National Institutes of Health. The MRI CBF data were processed using MANGO software developed by the Research Imaging Institute of the University of Texas Health Science Center at San Antonio. We thank Andrew Yackzan and Kelly Roberts for assisting with experiments.

## Data availability statement

The original contributions presented in the study are included in the article/supplementary material, further inquiries can be directed to the corresponding author. The gene amplicon sequence data generated as part of this study is available in the NCBI BioProject database (PRJNA903182).

## Ethics statement

The animal study was reviewed and approved by Institutional Animal Care and Use Committee at the University of Kentucky.

## Author contributions

LY, AB and A-LL designed research. LY, Y-HC, GC, SG, ZL, MS, AB, and A-LL conducted research. LY, Y-HC, GC, SG, ZL, XX, MS, TH, and A-LL performed statistical analysis. LY, Y-HC, GC, SG, ZL, XX, MS, TH, AE, AB, and A-LL wrote paper. LY, Y-HC, GC, SG, ZL, XX, MS, TH, AE, AB, A-LL had primary responsibility for final content. All authors contributed to the article and approved the submitted version.

## Funding

This research was supported by NIH grants RF1AG062480 (funded by NIA and ODS) to A-LL, NIH/NIDDK Training Grant T32DK007778 to LY, and NIH/NIA TRIAD grant training T32AG057461 to TH and NIH/NINDS R01NS103785 to AB. The funders had no role in study design, data collection and analysis, decision to publish or preparation of the manuscript.

## Conflict of interest

The authors declare that the research was conducted in the absence of any commercial or financial relationships that could be construed as a potential conflict of interest.

## Publisher’s note

All claims expressed in this article are solely those of the authors and do not necessarily represent those of their affiliated organizations, or those of the publisher, the editors and the reviewers. Any product that may be evaluated in this article, or claim that may be made by its manufacturer, is not guaranteed or endorsed by the publisher.
